# Translating Alcohol Research Into Practice

**Published:** 2015

**Authors:** Edith V. Sullivan, Antonio Noronha

**Affiliations:** Edith V. Sullivan, Ph.D., is a professor in the Department of Psychiatry and Behavioral Sciences, Stanford University School of Medicine, Stanford, California.; Antonio Noronha, Ph.D., is director of the Division of Neuroscience and Behavior, National Institute on Alcohol Abuse and Alcoholism, Rockville, Maryland.

**Figure:**
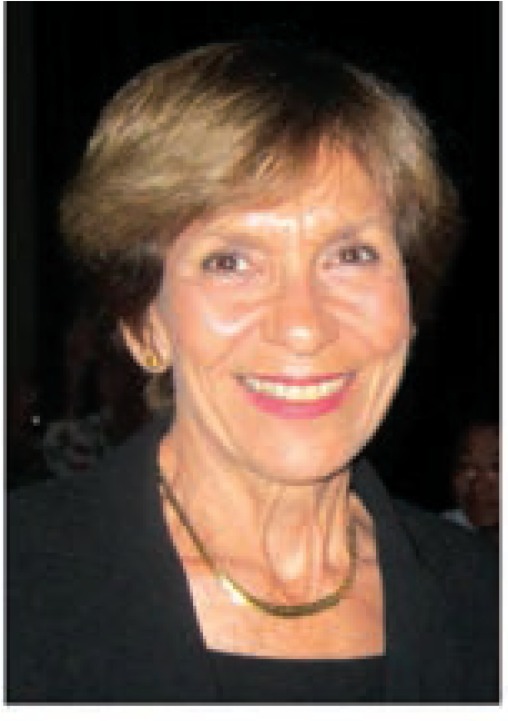

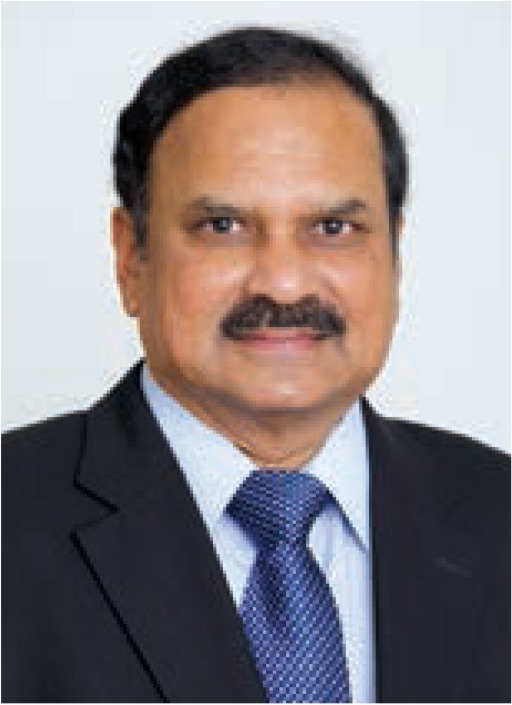


Translational research helps move basic science and clinical laboratory discoveries toward application in health and medicine. Through controlled experiments, basic scientists use animal models to reproduce disease characteristics caused by an agent—in this case, excessively high exposure to alcohol. Through systematic study and observation, clinical research scientists identify symptomatic and physiological characteristics that define a disease. These bidirectional, complementary approaches enhance basic and practical knowledge about the biological basis of disease and hold promise for creating thoughtful, scientifically motivated solutions for improving public health.

More than a decade ago, the National Institutes of Health (NIH) recognized the need for accelerating translation of research findings. In 2003, NIH developed the “Roadmap for Medical Research,” a collection of initiatives designed to find ways of motivating the translation of basic science into practice with the goal of transforming health care and outcomes. That vision was further crystallized in 2011 with the formation of the National Center for Advancing Translational Sciences (NCATS), which creates integrated translational and clinical research programs and fosters collaborations with community partners.

NIH’s attention to translational research demonstrates its quest to bridge the laboratory–clinic divide. In this issue, we examine how current research in the alcohol field is setting the stage for translating clinical research findings to alleviate physical damage and restore neuropsychological function in individuals with alcohol use disorder (AUD).

## Neuroadaptation: For Better and For Worse

A highly promising area is the translation of cognitive neuroscience discoveries to gain a basic-science understanding of behavioral and pharmacological treatment outcomes. Findings from structural magnetic resonance imaging (MRI) and functional MRI (fMRI) studies, both in active and in abstinent alcoholics, are leading to a better picture of cognitive pathways that underlie behavior change in AUD (see the article by Naqvi and Morgenstern). A more complete understanding might be achieved by tracking the condition of the brain’s communication networks through the cycle of alcoholism from sobriety to relapse and recovery. Indeed, anomalous brain circuitry is present in certain individuals with a genetic predisposition for alcoholism (see the articles by Kamarajan and Porjesz and by Cservenka and colleagues). Knowing more about these networks may enable us to identify people at high risk for developing an AUD. In earlier research, electrophysiological recordings obtained with scalp electroencephalography (EEG) first helped explain the genetic predisposition for AUD in individuals with a dense family history of alcoholism (see the article by Kamarajan and Porjesz). An extension of these studies could contribute to prevention and individualized treatment regimens.

Research using fMRI to examine brain network connectivity suggests that alcoholism may be related to problems in maintaining balance between opposing networks. As Fein and Cardenas explain, it may be possible to treat AUD by altering selective brain network function with focused magnetic stimulation applied transcranially and with the use of neurofeedback. Neuroimaging studies also have identified neuronal changes that may be associated with drinking relapse. Underlying the chronic relapse risk may be negative neuroplastic changes in the brain, caused by the cycle of continued alcohol abuse, repeated brief alcohol abstinence, and alcohol withdrawal.

In light of the multifactorial nature of AUD, we now know that factors such as stress affect brain circuitry in recovering alcoholics (see the article by Seo and Sinha). Hence, basic research through animal models of stress is critical for testing the effects of stress surmised from human studies. Lovinger and Kash review the effects of high alcohol exposure on two brain regions in a limbic pathway—the striatum and bed nucleus of the stria terminalis (BNST)—involved in long-term stress-related behaviors. This research should enhance our understanding of the mechanisms by which alcohol produces its long-lasting change in synaptic function (i.e., synaptic plasticity) and their potential as new treatment strategies.

Excessive alcohol consumption by pregnant women is particularly dangerous to the fetus. The timing of hazardous drinking occurring during fetal development dictates the locus and extent of neural and facial dysmorphology sustained, resulting in fetal alcohol spectrum disorders (FASD). Landmark studies of human FASD conducted by Riley and his colleagues and reviewed in their article (see Murawski et al.) also demonstrate how animal models can enhance the role of neuroimaging in human studies and lead to advances in FASD diagnosis and treatment. Animal models of FASD have yielded essential information about normal brain development and the temporal and dosage parameters of alcohol exposure that produce significant dysmorphology (see the article by Wang and Kroenke). In turn, this knowledge has provided suggestions for treatment.

## Translating Alcohol Research

Alcoholism is a complex human disorder with biomarkers for neural health dysfunction that are now detectable with neuroimaging methods. Clearly, alcohol researchers have led the way in applying these methods to identify—in the living brain—neural changes that occur with chronic, hazardous substance use. This accommodation of brain structure and function to the chronic presence of alcohol, that is neuroadaptation, seems to be either reversible or at least reduced with sustained sobriety. The promise of many of the functional imaging studies, then, is to identify neural circuits affected by and maladapted to alcohol, as well as those intact enough to undergo further neuroadaptation, but now in the direction of normalization. Knowledge about bidirectional neuroadaptation also might lead to the development of medications based on neural circuitry that expresses neuroplasticity (see the article by Johnson and Oslin) and identification of individuals with AUD who are treatment candidates because of their adequate neuroadaptive reserve. By fostering innovative collaborations across government, academia, industry, and the people we strive to treat, we can make the most of translational research findings and meet the growing need for specialized treatment (see the article by Batman and Miles). Research designs with both basic and clinical translational components hold promise for preventing and treating AUD and sustaining sobriety.
